# Electromyographic Assessment of Muscle Activity in Children Undergoing Orthodontic Treatment—A Systematic Review

**DOI:** 10.3390/jcm13072051

**Published:** 2024-04-02

**Authors:** Liliana Szyszka-Sommerfeld, Magdalena Sycińska-Dziarnowska, Mariangela Cernera, Luigi Esposito, Krzysztof Woźniak, Gianrico Spagnuolo

**Affiliations:** 1 Department of Maxillofacial Orthopaedics and Orthodontics, Pomeranian Medical University in Szczecin, Al. Powstańców Wielkopolskich 72, 70111 Szczecin, Poland; magdadziarnowska@gmail.com (M.S.-D.); krzysztof.wozniak@pum.edu.pl (K.W.); 2 Laboratory for Propaedeutics of Orthodontics and Facial Congenital Defects, Pomeranian Medical University in Szczecin, Al. Powstańców Wielkopolskich 72, 70111 Szczecin, Poland; 3 Department of Neurosciences, Reproductive and Odontostomatological Sciences, University of Naples “Federico II”, 80131 Napoli, Italy; mariangela.cernera@icloud.com (M.C.); luigi.esposito0995@gmail.com (L.E.); gspagnuo@unina.it (G.S.); 4 School of Dentistry, College of Dental Medicine, Kaohsiung Medical University, Kaohsiung 80708, Taiwan

**Keywords:** surface electromyography, muscle activity, orthodontic treatment, malocclusion, oral health, stomatognathic system, neuromuscular function, orofacial muscles

## Abstract

**Background**: Surface electromyography (sEMG) can provide an objective and quantitative image of the functional state of neuromuscular balance in the stomatognathic system. The objective of this systematic review is to examine current scientific evidence regarding the effects of orthodontic treatment on muscle electromyographic (EMG) activity in children. **Methods**: The search strategy included the PubMed, PubMed Central, Web of Science, Scopus, and Embase databases. The inclusion criteria were studies assessing EMG muscle activity in children undergoing orthodontic treatment compared with untreated children. The Cochrane risk-of-bias tool (RoB2) and the Newcastle–Ottawa Scale (NOS) were used to evaluate the quality of the studies. The quality of evidence assessment was performed using GRADE analysis. The PRISMA diagram visually represented the search strategy, as well as screening and inclusion process. **Results**: The search strategy identified 540 potential articles. Fourteen papers met the inclusion criteria. Six studies were judged at a low risk of bias. The certainty of evidence was rated as moderate to low, according to the GRADE criteria. Studies showed alterations in EMG muscle activity in children undergoing orthodontic treatment. **Conclusions**: Orthodontic treatment appears to affect muscle activity in children undergoing orthodontic treatment. However, the quality of evidence is low and, therefore, it is not possible to definitively state this effect. Further long-term studies are needed to confirm the findings of this review. Study protocol number in PROSPERO database: CRD42023491005.

## 1. Introduction

The primary aim of orthodontic treatment is to achieve ideal relationships between teeth both within and between arches by moving the position of teeth and by modifying the skeletal structures and growth of the craniofacial skeleton [1]. Orthodontic treatment is capable of improving the oral health-related quality of life of patients through its impact on occlusion, function, and esthetics [2,3]. Achieving neuromuscular balance at the end of orthodontic treatment is another very important objective that cannot be disregarded. A correlation appears to exist between neuromuscular function and the stability of occlusion [4]. In light of the above, it is not enough to rely exclusively on the classic structural and esthetic parameters used in orthodontics to evaluate the functional aspects of the stomatognathic system [1]. One point that has been emphasized by researchers is that neither a proper diagnosis of malocclusion nor any assessment of the results of orthodontic treatment should be based solely on clinical and cephalometric evaluations. Rather, they should also involve the use of surface electromyography (sEMG) [5]. sEMG helps ensure accurate diagnoses and monitor the functional impact of orthodontic treatment at different stages. This method can provide an objective and quantitative image of the functional state of neuromuscular balance in the stomatognathic system and serve as an important instrument for studying the relationship between the morphology and function of the oral-maxillofacial system [6,7,8,9].

sEMG is a non-invasive technique used to measure electrical activity. It does so by identifying electrical signals on the skin located over the superficial muscles [6,10,11]. The advantage of this instrument-based method is its non-invasiveness, simplicity, and accessibility. These have proven to be important factors in studies involving children [12]. However, the usefulness of sEMG in the study of the stomatognathic system is debatable due to a number of shortcomings of this method that may impair its clinical effectiveness. One of the main disadvantages of surface electromyography is its sensitivity to impedance imbalances, which may reduce the accuracy of the electromyographic (EMG) recordings. Various factors can undermine its validity, including problems with the reproducibility of sEMG due to technical artifacts (instrumental noise), anatomical variations, such as facial morphology, age, gender, and the thickness of the subcutaneous fat, as well as muscle cross-talk [7,13,14,15]. The introduction of standardized EMG protocols and indices to assess the activity of paired masticatory muscles allows for more reliable analyses [7,8,9].

A number of previous studies have shown the effect of functional orthodontics and orthodontics on masticatory muscle activity [16,17,18,19]. It should be pointed out that orthodontic treatment includes a wide range of methods and devices used that may affect muscles in different ways. The mechanism of orthodontic functional treatment involves changing the position of the upper and lower jaw, thereby enabling a shift in the position of the attached muscles to a certain extent, which in turn may lead to changes in the masseter muscle [20]. It has been demonstrated that various methods of orthodontic treatment, e.g., rapid palatal expansion (RPE), not only have an orthopedic effect on the maxillary complex, but also affect the functional activities of the masticatory muscles [21,22]. Changes in the masticatory muscle activity during treatment with fixed orthodontic appliances can be due to pain or to changes in the occlusal relationship between the maxillary and mandibular dentitions, thus providing new periodontal afferents that may influence the neuromuscular equilibrium [23]. This justifies EMG recordings of the masticatory muscles before, during, and after orthodontic treatment, with the aim of monitoring or assessing its effectiveness [7]. In addition, sEMG plays an important role in the diagnosis of facial muscles during orthodontic treatment due to its neuromuscular approach and the facial pain associated with the use of functional appliances [21]. EMG data on muscle activity can be recorded in different conditions, such as resting, chewing, sucking, swallowing, clenching, and contraction of the orofacial muscles [21,24,25,26].

The available literature includes many studies on the impact of various methods of orthodontic treatment on patients’ orofacial muscles, although no definite conclusions have been drawn so far [1,18,23,27,28,29,30]. In addition, existing papers differ in terms of their study populations, interventions, and outcome measures. A few of these studies have focused on orthodontically treated children [16,17,22,31,32]. Therefore, the objective of the present systematic review is to meticulously examine and consolidate current scientific evidence regarding the effects of orthodontic treatment on muscle activity in children based on sEMG. The main aim of this paper is to assess alterations in muscle electromyographic (EMG) activity in children undergoing orthodontic treatment when compared with untreated children.

## 2. Materials and Methods

Systematic Review registration statement: The review protocol was registered at the PROSPERO International Prospective Register of Systematic Reviews (https://www.crd.york.ac.uk/prospero, registered on 21 December 2023), registration No. CRD42023491005. When performing this systematic review, the authors followed the PRISMA (“Preferred Reporting Items for Systematic Reviews and Meta-Analyses”) guidelines [33] (Supplementary Tables S1 and S2). 

### 2.1. Search Strategy

In line with PICOS [34], the framework of the systematic review was as follows:

Population (P): Children between the ages of 6 and 16 undergoing orthodontic treatment. No restriction will be applied with regard to the type and severity of malocclusion, orthodontic method, or orthodontic appliances involved.

Intervention (I): orthodontic treatment using different methods or types of orthodontic appliances in children with malocclusion requiring orthodontic therapy.

Comparison (C): untreated control children.

Outcomes (O): Electromyographic analysis of alterations in muscle activity in children undergoing orthodontic treatment. An assessment of improvements in muscle function measured according to changes in muscle electrical potentials by means of surface electromyography—no restrictions will be applied to the type of muscle analyzed (muscles of the orofacial region) or the conditions/tests used.

Study design (S): randomized controlled trials (RCTs), controlled clinical trials (CCTs), and observational studies (including case-control and cohort studies).

The PICOS questions were as follows: “Does muscle electromyographic activity in children undergoing orthodontic treatment differ from muscle activity in untreated children?” and “What impact does orthodontic treatment have on EMG muscle activity in treated children compared with untreated control children?”

A search of the following databases was performed: PubMed, PubMed Central, Web of Science, Scopus, and Embase, using the following keywords: “surface electromyography” AND “muscle activity” AND “orthodontic treatment” OR “orthodontics” AND “children”. The final literature search was completed on 31 December 2023.

Two independent reviewers (L.S.-S. and M.S.-D.) conducted an exhaustive and unbiased literature search that did not take into account language or publication date. At the same time, references to the corresponding articles were retrieved manually, and an as comprehensive as possible search was conducted of the related literature. The search was repeated prior to the final analysis.

### 2.2. Eligibility Criteria

For this systematic review, the following inclusion criteria were applied:

- Study type: all types of controlled trials and observational studies on EMG muscle activity in children undergoing orthodontic treatment,
- Outcome of interest: electromyographic analysis of alterations in muscle activity in children undergoing orthodontic treatment,
- Object of the study: electromyographic assessment of muscle activity in orthodontically treated children compared with untreated children,
- Participants: human subjects—children between the ages of 6 and 16 undergoing orthodontic treatment.

The exclusion criteria were as follows: ineligible study design, e.g., case reports, literature or systematic reviews, animal studies, and unpublished data; ineligible interventions and outcome measures, e.g., studies concerning changes in EMG resulting from the surgical correction of malocclusion, studies assessing EMG muscle activity in individuals with malocclusion but without at the same time assessing EMG changes following orthodontic treatment, studies assessing EMG muscle activity in groups of orthodontically treated children without any comparison with a control group, and lack of effective statistical analysis; ineligible study populations, e.g., studies on adults, studies on children under 6 years, and children with cleft lips and/or palates and other craniofacial abnormalities.

### 2.3. Data Extraction

After removing duplicate publications, the titles and abstracts of the remaining studies were carefully reviewed by the first author (L.S.-S.), after which they were assessed by the second author (M.S.-D.), with the aim of identifying potentially eligible studies. Subsequently, the full texts of the selected papers were examined in depth and, based on pre-established inclusion and exclusion criteria, were either accepted or rejected. Only studies assessing muscle electromyographic activity in children undergoing orthodontic treatment compared with untreated children were considered for inclusion. Any uncertainties or ambiguities encountered during the process were resolved by the third author (G.S.). The following information was extracted from each study: the author(s), date of publication, study design, number, age and gender of the sample (characteristics of the participants), interventions (type of malocclusion, orthodontic method or type of orthodontic appliances used, as well as the duration/stage of the orthodontic treatment), outcome measures (including information regarding the EMG instrument, the type of muscles examined, conditions/tests, as well as the EMG signal analysis domain), and the principal findings. A reviewer (L.S.-S.) collected the results from each study and documented them in an Excel spreadsheet. In cases where the information was incomplete, the corresponding authors of the studies included in the review were contacted and asked to provide as many additional details as possible.

### 2.4. Quality Assessment

The authors evaluated the quality of the non-randomized studies included in the review using the Newcastle–Ottawa Quality Assessment Scale (NOS) [35]. The NOS application consists of assigning a maximum of one point (one star (*)) for each numbered item within the selection and outcome categories, except for the item comparability, in which a maximum of two stars (**) can be given. Therefore, according to NOS protocol papers are awarded stars for three different criteria, i.e., selection (worth a maximum of 4 stars), comparability (a maximum of 2 stars), and outcome (a maximum of 3 stars (***)), to earn a maximum score of 9 stars. The total score was attributed to the categories of “high risk of bias” (total score of 0–3), “moderate risk of bias” (total score of 4–6), and “low risk of bias” (total score of 7–9). Randomized controlled trials were assessed using version 2 of the risk of bias tool (RoB2), recommended by the Cochrane Collaboration [36]. The RoB2 tool assesses bias in five distinct domains: bias arising from the randomization process, bias due to deviations from intended interventions, bias due to missing outcome data, bias in the measurement of the outcome, and bias in the selection of the reported result. The judgments within each domain lead to an overall risk of bias of: “low risk of bias”, “some concerns”, or “high risk of bias”. The quality assessment was performed independently by two authors (L.S.-S. and M.S.-D.). All ambiguities were resolved by a third author (K.W.).

### 2.5. Certainty of Evidence

Studies were assessed for certainty of evidence using the Grading of Recommendations, Assessment, Development, and Evaluation (GRADE) scale [37]. The GRADE approach classifies the quality of evidence for each outcome by assessing the following domains: study design, risk of bias, inconsistency, indirectness, imprecision, publication bias, magnitude of the effect, dose–response gradient, and the influence of all plausible residual confounders. The quality of evidence was then classified as follows:

- High certainty: we are very confident that the true effect lies close to that of the estimate of the effect.
- Moderate certainty: We are moderately confident in the effect estimate. The true effect is likely to be close to the estimate of the effect, but there is a possibility that it is substantially different.
- Low certainty: Our confidence in the effect estimate is limited. The true effect may be substantially different from the estimate of the effect.
- Very low certainty: We have very little confidence in the effect estimate. The true effect is likely to be substantially different from the estimate of the effect.

Two authors (L.S.-S. and M.S.-D.) assessed the studies independently, and any discrepancies were resolved by consensus with a third author (G.S.).

### 2.6. Data Synthesis

A PRISMA diagram was generated to provide a visual representation of the entire search strategy as well as the subsequent screening and inclusion process. After a comprehensive review of the studies included in the review, the most significant data regarding the participant characteristics, interventions, outcome measures, and principal findings were tabulated using Microsoft Excel version 2016 spreadsheet software to display the results of each study. Finally, a narrative synthesis was performed after tabulating the results, describing the differences between the studies in terms of their methodologies, interventions, aims, and findings.

## 3. Results

**Figure 1:**
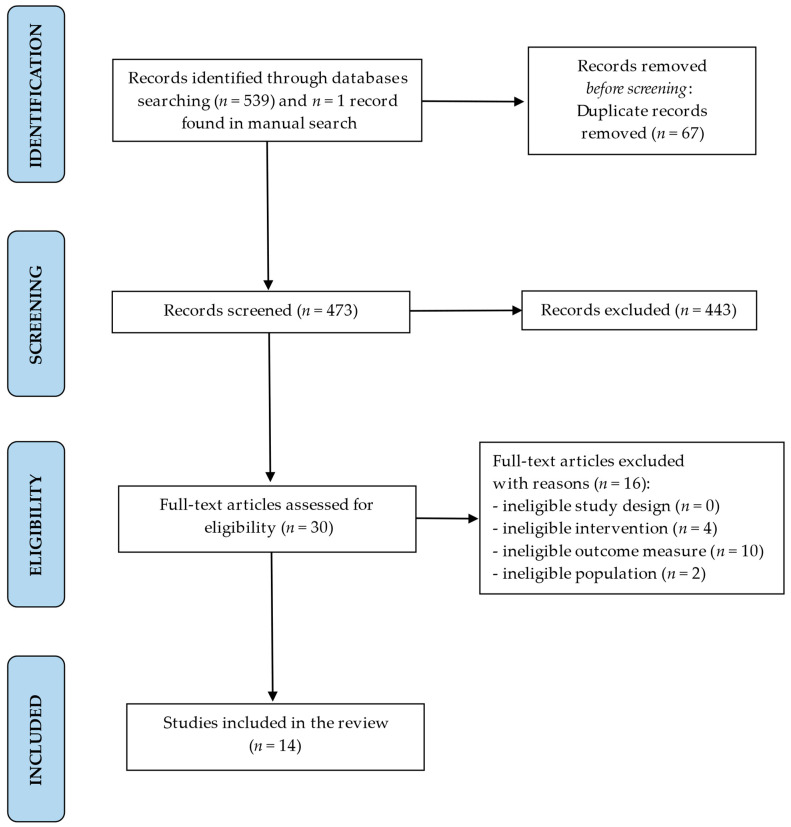
PRISMA flow diagram for the search strategy.

The search strategy identified 540 potential articles: 140 from PubMed, 326 from PubMed Central, 40 from Embase, 21 from Scopus, 12 from Web of Science, and 1 study identified by manual search. After removing 67 duplicates, a total of 473 articles were analyzed. Following title and abstract screening, 443 papers were excluded based on the predetermined inclusion and exclusion criteria. Of the remaining 30 articles, 16 were excluded because they were studies of ineligible interventions or outcome measures, or studies involving ineligible populations. Eventually, a total of 14 papers were included in the review. The entire process was schematized in the Prisma Flow Diagram (Figure 1, flow diagram). Table 1 displays the principal features of each study that were included in the review.

### 3.1. Results of the Quality Assessment

Based on the NOS and RoB2 tools, eight studies were judged at a moderate risk of bias [5,17,22,31,38,40,43,45] and six were judged at a low risk of bias [1,32,39,41,42,44] (Table 2).

### 3.2. Findings of Certainty of Evidence

The overall certainty of evidence was rated as “moderate” [38,44] to “low” [1,5,17,22,31,32,39,40,41,42,43,45] according to the GRADE criteria (Table 3). The outcomes assessed were changes in muscle activity by means of sEMG in children undergoing orthodontic treatment and untreated children. Furthermore, the outcomes were sub-grouped into randomized (RSs) and non-randomized (NRSs) studies.

### 3.3. Characteristics of the Study Groups (Age, Malocclusion, and Orthodontic Method/Orthodontic Appliances)

The studies included a total of 764 patients aged between 7.6 [31] and 16.15 [1].

Most of the studies concerned children with Class II malocclusions [1,17,31,38,40,41,43]. Five studies examined subjects with Class II malocclusions treated with functional appliances [17,31,38,41,43]. Satygo et al. [31] and Uysal et al. [43] assessed muscle activity in children undergoing functional treatment with a Pre-Orthodontic Trainer (POT). Similarly, Saccucci et al. [17] included participants undergoing treatment with a preformed orthodontic/functional device, while the patients in the studies conducted by Erdem et al. [38] and Petrović et al. [41] were treated with an activator. Masci et al. [1] included patients diagnosed with Class II malocclusion and treated with fixed multibracket appliances and use of Class II elastics. Ocak et al. [40] studied patients undergoing orthodontic treatment that included the use of utility arches.

Yuen et al. [45] divided the study participants according to the type of functional orthodontic appliances used in their treatment, namely, Bionator, Fränkel type I, or Fränkel type III.

Four studies concerned children with posterior crossbites treated with various orthodontic appliances [5,32,39,42]. Martín et al. [39] examined children who were treated with a Quad-Helix (QH) appliance. Similarly, Kecik et al. [5] compared children with functional posterior crossbite (FBXB) and children without malocclusion both before and after maxillary expansion treatment with a QH appliance. Piancino et al. [42] examined participants who had been treated with a customized functional appliance, known as a “Function Generating Bite”. Michelotti et al. [32] evaluated muscle activity in patients with unilateral posterior crossbite (UPXB) before and after rapid maxillary expansion (RME).

Spolaor et al. [22] studied patients with different types of malocclusion, such as patients with bilateral posterior crossbite (BPXB), UPXB, and children with an underdeveloped maxillary complex without crossbite (NOXB) treated with RME.

The focus of the study conducted by Wasinwasukul et al. [44] was children diagnosed with a deep bite. In this case, to correct their deep bite the children were treated using anterior bite planes fabricated from acrylic resin (ABP) or bi-laminate thermoplastic (TBP), designed to raise their bite.

### 3.4. Electromyographic (EMG) Muscle Activity

Muscle activity was reported by means of sEMG in different conditions, such as: (a) resting position [1,5,17,39,40,41], (b) clenching, including MVC (maximal voluntary clenching) in the inter-cuspal position and MVC on cotton rolls [5,17,31,32,38,39,40,41,43,44,45], (c) chewing [22,32,38,39,42], (d) swallowing [5,17,38,39,41,43], as well as other conditions, such as (e) whistling [38], (f) tightening the lips [40], (g) kissing, opening the mouth, and protrusion of the mandible [17].

Out of the 14 studies that were reviewed, 12 studies assessed the masseter [1,5,22,31,32,38,39,40,42,43,44,45] and 10 studies assessed the temporal muscles [1,5,22,31,32,38,39,43,44,45], while the orbicularis oris muscles were evaluated in 4 studies [17,38,40,43]. Other analyzed muscles included the suprahyoid (SH) [39], mental [43], digastric (DA), and sternocleidomastoid (SC) muscles [1,5].

Studies showed alterations in EMG muscle activity in children undergoing orthodontic treatment compared with untreated children [1,5,17,22,31,32,38,39,40,41,42,43,44,45].

Following functional treatment of Class II malocclusion subjects, a significant increase in EMG activity of the masticatory [31,38,41] and perioral [17,43] muscles was observed. Satygo et al. [31] reported that functional treatment with the POT significantly increased the EMG activity of the TA and MM muscles during clenching in patients with CII/1 malocclusion. Similarly, Uysal et al. [43] showed that POT treatment increased OO activity during sucking and clenching, but it remained unchanged during swallowing. Saccucci et al. [17] noted a significant increase in the EMG activity of the lower OO muscle at rest and of the upper OO muscle during protrusion of the mandible after treatment with a preformed functional device. Erdem et al. [38] reported considerable differences in the EMG activity of the TA and MM during maximal clenching and chewing and in the OO activity during whistling after 12 months of treatment with Andresen activators. Petrović et al. [41] found that in all measured positions of the mandible with CII/2 malocclusion, the electrical activity of the TA and MM muscles was lowest at baseline and increased during the first year of treatment with an activator, while at the end of the treatment it declined somewhat and was close to the approximate values in normal occlusion.

Similarly, significant differences in muscle activity were observed after treating patients with Class II malocclusion with fixed orthodontic appliances [1,40]. Masci et al. [1] found that the EMG activity of the TA muscles was significantly higher in treated subjects under open eye conditions, and Ocak et al. [40] showed that upper incisor protrusion induced a significant increase in the SOO activity. This increase remained stable after retention.

On the other hand, Yuen et al. [45] observed that although the muscles of untreated children also showed shifts in mean frequency toward lower values as a function of time, a greater downward shift in mean frequency occurred in those subjects treated with functional appliances.

Studies also demonstrated significant changes in masticatory muscle activity after the correction of transverse types of malocclusion [5,22,39,42]. Martín et al. [39] found that orthodontic treatment with a QH appliance improved the functional capacity of the masticatory muscles during mastication: crossbite side MM activity showed significantly higher EMG values after treatment and retention, indicating that the increased electrical activity achieved after treatment remained stable. During clenching, the initial predominance of TA over MA activity reversed after retention, leading to normalization of the activity of these muscles. Kecik et al. [5] showed significant differences between the groups in terms of TA and MM muscle activity during rest, swallowing, and clenching. In their study, Piancino et al. [42] observed that the correction of UPXB with a “Function Generating Bite” induced a normal-like coordination of MM muscle activity, together with a significant reduction of the reverse chewing patterns. Spolaor et al. [22] discovered a relationship between the correction of a maxillary transverse discrepancy with RME and the normalization of muscle activation patterns comparable to healthy subjects for both TA and MM. On the contrary, Michelotti et al. [32] concluded that RME-based treatment of UPXB did not result in more symmetrical activity of the assessed muscles.

Wasinwasukul et al. [44] reported that at maximum clenching, masticatory muscle activity immediately dropped significantly, but then returned to baseline values and was the same as the control group at 1–3 months.

## 4. Discussion

This systematic review presented the relevant findings in the literature on the impact of orthodontic treatment on muscle electromyographic activity in children. The review covered a total of fourteen studies examining changes in EMG activity of the orofacial muscles in orthodontically treated children compared with untreated children. Among these, six studies were judged at a “low risk of bias” [1,32,39,41,42,44] based on the NOS and RoB2 tools. The overall quality of evidence of the studies ranged from “low” [1,5,17,22,31,32,39,40,41,42,43,45] to “moderate” [38,44], according to the GRADE criteria.

In general, studies showed alterations in muscle electrical activity in children undergoing orthodontic treatment [1,5,17,22,31,32,38,39,40,41,42,43,44,45]. In addition, the kinds of changes observed in EMG muscle activity varied depending on the muscle and task studied, as well as on the type of malocclusion involved and the method of orthodontic treatment/type of orthodontic appliances used. However, because of the low quality of evidence of the literature resulting from the limited number of randomized studies and other methodological limitations, these findings need to be carefully considered. It should be pointed out that the low quality of evidence and the high heterogeneity among studies in terms of their study designs, types of interventions (different orthodontic methods/appliances involved), and/or standardization of EMG protocols could lead to an overestimation of the results.

In assessing orthodontically treated children using sEMG, the tasks that were most frequently analyzed included rest position and clenching, swallowing, and chewing tasks, while the most frequently analyzed muscles were the MM, TA, and OO muscles [1,5,17,22,31,32,38,39,40,41,42,43,44,45]. The most frequently applied orthodontic methods were the functional treatment of Class II malocclusions [17,31,38,41,43] and the treatment of transverse types of malocclusions using QH appliances [5,39] or RME [22,32]. In particular, studies have shown that EMG activity of the masticatory [31,38,41] and perioral [17,43] muscles was lower before treatment in the case of children with Class II malocclusion than was the case with a group of controls. Following treatment, a significant increase in EMG muscle activity was observed in the Class II malocclusion subjects. Such an increase in muscle electromyographic activity signifies a possible impact of orthodontic treatment in normalizing masticatory and perioral muscle function. In this context, care should be taken when proposing functional growth modification in children with pre-existing high muscle activity levels. There is some limited evidence that the effect of functional appliances on orofacial tissues may be predicted by pretreatment muscle thickness and bite force, both of which are associated with masticatory muscle activity. Greater dentoalveolar changes in response to functional appliances have been observed in patients with thinner masseter musculature and a lower bite force [46,47]. sEMG also demonstrated a relationship between the correction of a maxillary transverse discrepancy and the normalization of a masticatory muscle’s activation patterns [5,22,39,42]. Furthermore, the results indicate that orthodontic treatment may cause more symmetrical MM muscle activity [39] and reduce the high frequency of the reverse chewing cycle in children with UPXB [42], thus indicating the restoration of the coordination of bilateral MM muscle activity and inducing a possible favorable change in the neuromuscular control of chewing [39,42,48]. These findings may be particularly important in early treatment of children diagnosed with UPXB with mandibular lateral shift [5,39].

Normal jaw function depends on a harmonious relationship between the different components of the stomatognathic system. This harmonious relationship may be disrupted in children with malocclusion, which may affect the normal growth and function of the jaw [49]. From a clinical point of view, it is vital to note that orthodontic treatment seems to affect muscle activity in children with malocclusion. These findings are especially important in correction of posterior crossbites. Previous studies emphasized that the occlusal condition developed in response to UPXB could generate an inhibitory–protective reflex over masticatory muscles to avoid injury of the structures of the stomatognathic system that would disappear after stable occlusion was achieved [50,51]. Other researchers found a correlation between occlusal stability and elevator muscle function, likely based on feedback mechanisms from periodontal pressoreceptors [52,53].

It should also be pointed out that nowadays, the high prevalence of malocclusion affects greater demand for orthodontic treatment, which in turn means that this kind of oral treatment plays an important role in maintaining oral health [6,54]. Correction of malocclusion can not only improve occlusal relationships and facial esthetics in patients but can also mitigate or improve the psychological feelings of inferiority experienced by patients with malocclusion [6,55,56,57]. In this context, we should remember that a posterior malocclusion, such as UPXB, is more likely to affect oral functions, especially chewing. These types of malocclusion destabilize normal tooth occlusion and jaw muscle orientation/alignment, which can severely affect normal chewing function by reducing the food grinding efficiency. Although orthodontic correction of all types of dental malocclusion has significant esthetic and psychological benefits, treating posterior malocclusion in particular could be key to restoring normal chewing function and improving the functional capacity of the muscles, as presented in the included studies [39,42,54,55].

Furthermore, when interpreting the study results on the impact of orthodontic treatment on muscle activity, it is important to remember that many factors, such as stress and other psychological factors during treatment, can also affect muscle function. Previous studies have shown higher masticatory muscles’ activity in people with high levels of stress [58]. Perceived stress also seems to be associated with changes in muscular asymmetry [59]. On the other hand, according to many studies, there is a correlation between temporomandibular disorders (TMD) and variables such as parafunctional habits and perceived stress [60,61]. In light of these considerations, it should be emphasized that most of the included studies excluded patients with clinical signs or symptoms of TMD [1,32,38,39,40,44] and those reporting oral parafunctions [17,32,40,43,44].

We used sEMG to assess muscle activity in children undergoing orthodontic treatment in this study. It is well known that the muscles of the orofacial region have a considerable impact on the development of dentition and occlusion formation, as well as on occlusal stability. The lack of muscle balance could compromise the stability of the results achieved by orthodontic treatment and could require the endless use of retainers for retention purposes [1]. In this context, it has been pointed out that research on the electromyographic activity of orofacial muscles can be useful in everyday clinical practice, especially when distinct skeletal discrepancies are present before, during, and after orthodontic treatment, as sEMG not only allows for monitoring or evaluating its effectiveness, but can also be helpful in retention planning [1,41].

To sum up, sEMG can provide an objective and quantitative assessment of the functional state of neuromuscular balance of the stomatognathic system by measuring muscle electrical potentials. In clinical orthodontics, surface electromyography has been used to evaluate the impact of occlusal conditions on the neuromuscular stability of the stomatognathic system [8,9,26] and to evaluate, from a functional point of view, the efficacy of various forms of orthodontic treatment [1,5,62,63]. However, when analyzing the study results, we should remember that the usefulness of sEMG in the study of the stomatognathic system is still debatable. Researchers emphasize the fact that physiological variables, such as age, sex, skeletal morphology, and psychological factors, may affect the validity of sEMG [1,13]. In addition, the precision of EMG outcomes depends, to a great extent, on a number of technical issues, such as the positioning of the electrodes, signal processing, as well as the particular hardware and software used [7,14]. The most common solution to inconsistencies in impedance, which affects the reliability of sEMG, is adequate quantitative electromyographic analysis involving normalization procedures. The normalization of sEMG results requires their conversion into quotient indices. Normalized EMG data will offer insights into how occlusion affects neuromuscular activity, while disregarding individual variations, such as anatomical variances, the physiological and psychological state of a patient, and other factors [7,64]. In this context, it is important to note that in the studies included in this review, EMG recordings of the subjects were performed using a variety of EMG devices with different technical parameters, while a variety of parameters were used to analyze the electromyographic signals in children. Data processing was performed in the amplitude domain with normalized [22,32,44] or non-normalized data [1,5,17,31,38,39,40,41,42], and in the frequency domain [43,45].

This systematic review includes a number of limitations that should be acknowledged. (a) Only six studies were judged at a low risk of bias [1,32,39,41,42,44] based on the NOS and RoB2 tools, and the overall quality of evidence of the studies ranged from “low” to “moderate”, according to the GRADE criteria. (b) In a few of the included studies, only a small number of children were enrolled [17,38,40,43,45]. (c) Differences in the types of malocclusion studied and their severity, as well as differences in the control groups that may have comprised normo-occlusive children [5,17,22,31,32,39,40,41,42,45] or untreated subjects with malocclusion [1,31,38,43,44], may affect the results. (d) It should also be pointed out that orthodontic treatment covers a wide range of methods and devices used that may affect muscles in different ways. (e) The use of various parameters to analyze the EMG signal, the lack of normalization protocols, as well as many other factors, such as physiological, psychological, and technical factors, may have also impacted the results of some of the studies included in this review. As a consequence, considering the abovementioned limitations, further studies are needed to improve the evidence on this topic.

## 5. Conclusions

This systematic review offers a comprehensive evaluation of muscle activity in children undergoing orthodontic treatment using surface electromyography. Alterations in EMG muscle activity were observed in orthodontically treated children compared with control groups during various tasks. However, the low quality of evidence in the literature (ranging from “low” to “moderate” according to the GRADE criteria) and the high heterogeneity among studies may lead to an overestimation of the results.

Nevertheless, based on the findings and within the limitations of the study, the following conclusions can be drawn. Orthodontic treatment appears to affect muscle activity in children undergoing orthodontic treatment. However, the quality of evidence is low and, therefore, the effect remains uncertain. Therefore, to avoid overestimating the effectiveness of orthodontic treatment on muscle function, further long-term studies are needed to enhance evidence on this topic. Specifically, larger and more robust high-quality studies are required to either refute or confirm the findings of this review.

## Figures and Tables

**Table 1:** Characteristics of the study interventions.

Authors, Year	Study Participants (Number, Age, Malocclusion, Orthodontic Treatment)	Outcomes	Results
Erdem et al., 2009 [38]	*n* = 25 subjects with CII/1 malocclusions who were randomly assigned to either a treatment group (*n* = 15; 9 girls, 6 boys; mean age 11.3 ± 1.1 years) or a control group (*n* = 10; 4 girls, 6 boys; mean age 11.0 ± 1.3 years). The subjects in the treatment group were treated with activators and the subjects in the control group were untreated.	The EMG recordings of the TA and MM muscles during clenching, chewing, and swallowing and the EMG activity of the OO muscle during whistling were obtained at the start of the study and 12 months later. The EMG recordings were performed with a Disa Neuromatic 2000 electromyograph device (Dantec DISA, Scovlunde, Denmark).	The EMG activity of the TA and MM muscles during clenching, chewing, and swallowing increased significantly in both groups, especially in the TA muscle during maximal clenching in the treatment group (*p* < 0.001). The EMG activity in the OO muscles during whistling increased significantly in the treatment group. All between-group EMG differences were statistically significant (*p* < 0.001 for the electrical activity of the masticatory muscles during maximal clenching and chewing, and *p* < 0.05 for the EMG activity of the OO muscles during whistling), except in the case of the EMG activity of the TA and MM muscles during swallowing (*p* > 0.05).
Kecik et al., 2007 [5]	*n* = 35 patients (20 girls, 15 boys) with FPXB at a mean age of 10.6 ± 1.4 years consisted of the experimental group; the control group consisted of 31 normo-occlusive subjects (18 girls, 13 boys) at a mean age of 9.8 ± 1.6 years. The subjects in the FPXB group were treated with the QH appliance.	The EMG activity of the TA, MM, SC, and DA muscles of both sides was recorded during rest, swallowing, and maximum clenching. The data were collected at 1 time point in the controls, and before treatment and 6 months after treatment (3 months of expansion and 3 months of retention) in the FPXB group.	In the FBCX group, TA and MM activity was significantly higher on the crossbite side at rest before treatment (*p* < 0.001). After treatment, differences between the two sides had been eliminated and no significant divergences between the FPXB and control groups were observed (*p* > 0.05). Significant differences were found between both groups during clenching (*p* < 0.001). EMG values in the TA muscle were significantly higher on the crossbite side before treatment (*p* < 0.001). The right and left sides of the control group showed no significant difference during clenching (*p* > 0.05). MM muscle activity was considerably higher on the non-crossbite side before treatment, and the difference between the groups was significant (*p* < 0.001). Following treatment, no significant differences were observed between the groups in terms of MM activity (*p* > 0.05).
Martín et al., 2012 [39]	*n* = 25 children (10 boys, 15 girls), aged 10 to 14 years old diagnosed with UPXB and functional mandibular lateral shift. *n* = 30 age-matched children with normal occlusion (15 boys, 15 girls) served as a control group. Orthodontic therapy consisted of expansion with the QH appliance.	The EMG activity of the TA, TP, MM, and SH muscle areas was evaluated at rest and during swallowing, mastication, and clenching. The study was performed using an EM2 electromyograph (K6-I Diagnostic System, Myotronics-Noromed, Kent, WA, USA).	At the beginning of the study, EMG activity in non-crossbite side TA during swallowing was considerably higher in a UPXB group than in controls (*p* < 0.01). During clenching, EMG activity in crossbite side MM was lower in the UPXB group (*p* < 0.05). After treatment, crossbite side TA and MM activity increased significantly (*p* < 0.001) and remained stable after retention. A significant reduction in the resting EMG activity of crossbite side TA (*p* < 0.001) and MM (*p* < 0.05) was noted post-treatment. During mastication, MM activity increased significantly (*p* < 0.001), and its asymmetry was corrected post-treatment.
Masci et al., 2023 [1]	This study enrolled 30 patients (20 females, 10 males, mean age: 15.78 years) with a CII/1 malocclusion treated with fixed multibracket appliances. A group of 30 subjects (19 females, 11 males; mean age: 16.15 years), selected among subjects with CII/1 malocclusion who underwent no treatment, served as the control group.	The EMG activity of the MM, TA, DA, and SC muscles of both sides was examined in closed and open eyes conditions. The EMG activity was recorded with an eight-channel K7 system (Myotronic Inc.; Seattle, WA, USA).	The EMG activity of the TA muscles was significantly higher in treated subjects compared to untreated patients in the open eyes condition (*p* < 0.05).
Michelotti et al., 2019 [32]	*n* = 29 children with UPXB (UPXB group, 13 boys, 16 girls; mean age 9.6 ± 1.6 years) and *n* = 40 UPXB-free controls (control group, 17 boys, 23 girls; mean age 10.5 ± 1.1 years) were recruited. The UPXB group was treated with RME.	The EMG activity of the left and right TA and MM muscles was recorded during MVC in intercuspal position, and MVC in intercuspal position on cotton rolls and chewing. In the UPXB group, data were collected before treatment (T0), after the correction of the UPXB (T1), and 6 months later (T2). A wireless EMG device (TMJOINT, BTS SpA, Garbagnate Milanese, Italy) was used.	Before treatment, both groups were characterized by asymmetric activity of the TA and MM muscles, with no differences between groups (*p* > 0.05). The treatment effected a decrease in EMG muscle activity (*p* = 0.040) and a more asymmetric pattern of muscle activation during chewing after correcting crossbite (*p* = 0.040), which returned to values similar to baseline at T2 (all *p* > 0.05).
Ocak et al., 2022 [40]	*n* = 20 patients (8 girls, 12 boys; mean age 10.29 ± 0.90 years) with CII/2 malocclusion were selected for the study group. *n* = 15 patients (5 girls, 10 boys; mean age 10.56 ± 1.06 years) with Angle Class I malocclusion were recruited as controls. Upper incisors were protruded with utility arch in the study group.	The EMG activity of the SOO and left and right MM was recorded during the rest position, tightening the lips, and clenching teeth, with a Biopac MP150 sEMG device (Biopac Systems Inc., Goleta, CA, USA), before and after upper incisor protrusion and at the 6-month retention.	A significant change over time in SOO (*p* < 0.001) and in both right (*p* < 0.05) and left MM (*p* < 0.01) m-EMG in a group of treated patients was observed. In the CII/2 group, SOO m-EMG values increased following upper incisor protrusion (*p* < 0.001), and this increase remained stable (*p* < 0.05). MM m-EMG measurements decreased after protrusion (*p* < 0.01) and then increased significantly after retention (*p* < 0.05). No differences were observed between the groups following protrusion, and considerable differences were noted in left MM m-EMG at the end of the retention (*p* < 0.05).
Petrović et al., 2014 [41]	The sample consisted of 100 subjects of both sexes, divided into the control group (*n* = 30, mean age 11.23 ± 1.56 years) with neutral dental arches and Class I occlusion, and the study group (*n* = 70, mean age 11.68 ± 1.21) of patients with CII/2 malocclusions treated with an activator.	The EMG recordings of the TA and MM muscles were conducted in the physiologic rest position, central mandible occlusion, and during MVC and saliva swallowing prior to treatment, after one year of the orthodontic treatment, and after the treatment with an activator.	There was no significant difference in the left TA muscle between the groups, while in the right TA muscle the significant difference was noted in all positions at the beginning of the treatment (*p* < 0.05). Intergroup analysis of the EMG activity of the MM muscles showed significant differences in both left and right muscles in all positions (*p* < 0.05), except CO. The results for the first year of treatment showed an increase in electrical activity in the TA and MM muscles (*p* < 0.05).
Piancino et al., 2016 [42]	*n* = 50 children (mean age 9.1 ± 2.3 years) with UPXB and *n* = 20 children (mean age: 9.5 ± 2.6 years) with normal occlusion were selected for the study. Each patient was treated with the functional appliance “Function Generating Bite”.	The mandibular motion and the muscle activity of the MM muscles during chewing were simultaneously recorded, before and after correction of UPXB, after a mean treatment time of 7.3 ± 2.4 months plus the retention time of 5–6 months, using a multichannel EMG amplifier (a part of the K7-I WIN Diagnostic System K7-I; Myotronics, Tukwila, WA, USA).	Before treatment, the difference between electromyography envelope peaks in treated patients was less than in controls (*p* < 0.01) and increased significantly after treatment (*p* < 0.05), reaching values close to the reference normal value.
Saccucci et al., 2011 [17]	*n* = 13 patients (9 males, 4 females; mean age 9.0 ± 1.5 years) with Class II malocclusion and deep bite treated with an orthodontic/functional device (Occlus-o-Guide™Ortho-Tain Inc., Toa Alta, Puerto Rico). *n* = 15 children (9 males, 6 females; mean age 9.5 ± 0.8 years) with normal occlusion were recruited as a control group.	The electrical potentials of the OO muscle were investigated by EMG (Bio-pak EMG, BIOEMG 800™, Bio research Assoc. Inc., Goleta, CA, USA), during the rest position, kissing, swallowing, opening of mouth, clenching of teeth, and protrusion of the mandible at T0 (before therapy for the treated group), and after three (T1) (only for the treated group) and six (T2) months of treatment for both groups.	Before treatment, except when swallowing, the EMG activity of the lower OO muscle was lower in treated patients compared to a control group, with significant differences noted at rest and during mandibular protrusion (*p* < 0.05). A significant increase in muscle activity was observed in the lower OO muscle at rest in the treated group from T0 to T1 (*p* = 0.004). A significant increase in the activity of the upper OO muscle during protrusion of the mandible was observed from T1 to T2 (*p* = 0.004). No significant differences in EMG activity were observed in the control group during the follow-up (*p* > 0.05). The muscular contractility of the treated patients was at a similar level to the control group at T2.
Satygo et al., 2014 [31]	*n* = 36 CII/1 malocclusion patients (mean age 7.6 ± 1.3 years) composed the treated group and wore the POT functional appliance. *n* = 22 children with a similar age and malocclusion composed the untreated controls. *n* = 20 children with no dental malocclusion participated as normal controls.	The EMG activity of the right and left MM and TA muscles at clench was recorded using an electromyograph (DuoTrode; Myotronics Inc., Seattle, WA, USA) before and after 12 months of treatment.	Children in the treatment group reported a significant bilateral increase in TA and MM electrical activity (*p* < 0.001). After treatment, they recorded EMG values similar to those measured in normal controls, whereas the values noted for the untreated controls remained lower than those recorded at the beginning of the study.
Spolaor et al., 2020 [22]	*n* = 53 children (26 girls and 27 boys) divided into 2 groups: *n* = 43 patients with malocclusion and *n* = 10 healthy controls. *n* = 10 patients with BPXB, *n* = 15 with UPXB, and *n* = 18 with NOXB were treated with RME.	The EMG activity of the right and left MM and TA muscles during chewing tasks was collected using the 8-channel Free EMG system, 1000 Hz (BTS Bioengineering, Quincy, MA, USA), before and after RME application and 3 months after removal.	The EMG measurements before treatment revealed significant differences in muscle activity in terms of mean activation and occurrence during chewing tasks in the case of all malocclusions and when compared with a control group. In particular, symmetrical malocclusions (BPXB and NOXB) were characterized by symmetrical and similar muscle function, while patients affected by UPXB presented with lower activity in terms of PoE and earlier activation with regard to PPoE on the contralateral side. The EMG activity in the case of BPXB and NOXB increased slightly compared to the control group. Three months after RME removal, the activity of all the muscles had decreased in terms of PoE, with the exception of RM in UPXB patients. In terms of PPoE, the biggest improvement was observed in NOXB, with a significant reduction occurring in the RM, LT, and LM muscles.
Uysal et al., 2012 [43]	*n* = 20 patients (10 boys, 10 girls; mean age: 9.8 ± 2.2 years) with a CII/1 malocclusion and incompetent lips. Patients were treated with POT (Myofunctional Research Co., Queensland, Australia). *n* = 15 subjects (6 boys, 9 girls; mean age: 9.2 ± 0.9 years) with untreated CII/1 malocclusions were used as a control group.	The EMG recordings of the TA, mental, OO, and MM muscles during clenching, sucking, and swallowing were performed in the treatment group at the beginning and at the end of the POT therapy (mean treatment period: 7.43 ± 1.06 months). Follow-up records of the control group were taken after 8 months of the first records. The EMG activity was taken using the Biopac-MP150 unit (BIOPAC Systems Inc., Goleta, CA, USA).	During the course of POT treatment, the electrical activity of the TA (*p* < 0.001), mental (*p* < 0.05), and MM (*p* < 0.001) muscles decreased significantly, while OO activity (*p* < 0.01) increased during clenching when compared with a control group. During sucking, the EMG activity of the TA muscle decreased in the treatment group and remained unchanged in the controls (*p* < 0.05). The EMG activity of the OO muscle increased in the treatment group during sucking (*p* < 0.05). Intergroup comparisons of the EMG activity of the MM muscles showed significant differences during swallowing (*p* < 0.01) and sucking (*p* < 0.01).
Wasinwasukul et al., 2022 [44]	*n* = 66 children (35 boys and 31 girls) aged 9–13 years were randomly assigned to the ABP, TBP, or untreated control groups. The treated group of children had a deep bite.	The EMG activity of the TA and MM muscles was assessed at rest and during clenching via an 8-channel BioEMG III and BioPAK Measurement System (BioResearch, Inc., Brown Deer, WI, USA) before, immediately after appliance insertion, and after 2 weeks and 1, 3, and 6 months of treatment.	No statistical differences between two treatment groups and a control group during maximum clenching, in terms of the %MVC of all muscles before treatment (*p* ≥ 0.05), were observed. Immediately after appliance placement, MM activity was significantly lower in the ABP group compared with the TBP group (*p* < 0.05). At 1 month, the %MVC vales of all muscles in both treatment groups were not significantly different from the control group, with the exception of the TA of the TBP group (*p* < 0.05). From 3 months onward, no significant differences were observed between any of the groups in terms of the %MVC of any of the muscles (*p* ≥ 0.05).
Yuen et al., 1990 [45]	*n* = 18 children (9 boys and 9 girls) aged between 9.6 and 10.7 were divided into 3 groups receiving either Bionator, Fränkel type I, or Fränkel type III therapy. A fourth group consisting of *n* = 6 children (3 boys and 3 girls) aged 10.1 ± 0.8 years who underwent no treatment served as a control.	The EMG recordings of the TA and MM muscles were performed during MVC in the position of maximum intercuspation before and after 3, 6, and 12 months of therapy.	There was a greater downward shift in mean frequency that occurred in those subjects treated with functional appliances than untreated children. Changes in mean frequency in children treated with Bionator and Fränkel type I appliances were greater than in those treated with the Fränkel type III appliance.

EMG—electromyographic; TA—temporal anterior; TP—temporal posterior; MM—masseter; OO—orbicularis oris; SOO—superior orbicularis oris; DA—digastric; SH—suprahyoid; SC—sternocleidomastoid; CII/1—Class II division 1 malocclusion; CII/2—Class II division 2 malocclusion; QH—Quad-Helix appliance; RME—rapid maxillary expansion; MVC—maximum voluntary contraction (clenching); m-EMG—maximum contraction electromyography; CO—mandibular central occlusion; FPXB—functional posterior crossbite; UPXB—unilateral posterior crossbite; BPXB—bilateral posterior crossbite; NOXB—underdeveloped maxillary complex without crossbite; POT—Pre-Orthodontic Trainer; PoE—Peak of Envelope; PPoE—Position of Peak of Envelope; ABP—anterior bite planes fabricated from acrylic resin; TBP—anterior bite planes from bi-laminate thermoplastic.

**Table 2:** The quality assessment of the studies included.

**The Quality Assessment of the Non-Randomized Studies (NOS)**						
**Authors,** **Year**	**Selection**		**Comparability**		**Outcome**	**Total Score**
Kecik et al., 2007 [5]	**		**		**	6
Martín et al., 2012 [39]	***		**		**	7
Masci et al., 2023 [1]	***		**		**	7
Michelotti et al., 2019 [32]	***		**		**	7
Ocak et al., 2022 [40]	**		**		**	6
Petrović et al., 2014 [41]	***		**		**	7
Piancino et al., 2016 [42]	***		**		**	7
Saccucci et al., 2011 [17]	**		**		**	6
Satygo et al., 2013 [31]	**		**		**	6
Spolaor et al., 2014 [22]	**		**		**	6
Uysal et al., 2012 [43]	**		**		**	6
Yuen et al., 1990 [45]	*		**		**	5
**The Quality Assessment of the Randomized Studies (RoB2)**						
**Authors,** **Year**	**Randomization process**	**Deviations from the intended interventions**	**Missing outcome data**	**Measurement of the outcome**	**Selection of the reported results**	**Overall**
Erdem et al., 2009 [38]	Some concerns	Low risk	Some concerns	Some concerns	Some concerns	Some concerns
Wasinwasukul et al., 2022 [44]	Low risk	Low risk	Low risk	Low risk	Low risk	Low risk

NOS—Newcastle–Ottawa Quality Assessment Scale; RoB2—the Cochrane risk-of-bias tool version 2. The NOS application consists of assigning a maximum of one point (one star (*)) for each numbered item within the selection and outcome categories, except for the item comparability, in which a maximum of two stars (**) can be given. Therefore, according to NOS protocol, papers are awarded stars for three different criteria, i.e., selection (worth a maximum of 4 stars (****)), comparability (a maximum of 2 stars (**)), and outcome (a maximum of 3 stars (***)) to earn a maximum score of 9 stars. The total score was attributed to the categories of “high risk of bias” (total score of 0–3), “moderate risk of bias” (total score of 4–6), and “low risk of bias” (total score of 7–9). Based on the RoB2, the judgments within each domain led to an overall risk of bias of: “low risk of bias”, “some concerns”, or “high risk of bias”.

**Table 3:** GRADE summary of findings.

Outcomes	Impact	Number of Participants (Studies)	Certainty of Evidence (GRADE)	Comments
Muscle activity RSs	Significant impact reported in two studies.	91 (2 RS)	MODERATE ^a^ (downgraded by 1 level due to risk of bias)	Orthodontic treatment may affect muscle activity.
Muscle activity NRSs	Significant impact reported in eleven studies.	673 (12 NRS)	LOW ^b^	Orthodontic treatment may affect muscle activity.

GRADE—Grading of Recommendations, Assessment, Development, and Evaluation. “High certainty”—we are very confident that the true effect lies close to that of the estimate of the effect. “Moderate certainty”—we are moderately confident in the effect estimate: the true effect is likely to be close to the estimate of the effect, but there is a possibility that it is substantially different. “Low certainty”—our confidence in the effect estimate is limited: the true effect may be substantially different from the estimate of the effect. “Very low certainty”—we have very little confidence in the effect estimate: the true effect is likely to be substantially different from the estimate of the effect. ^a^ Certainty initially rated as “high” (the body of evidence contributing to an outcome consists of randomized studies, RSs). ^b^ Certainty initially rated as “low” (a body of evidence consisting of observational or non-randomized studies, NRSs).

## Data Availability

All data are available in the studies included in the review and were discussed in the present manuscript.

## Supplementary Materials

The following supporting information can be downloaded at: https://www.mdpi.com/article/10.3390/jcm13072051/s1; Table S1: PRISMA 2020 abstract checklist. Table S2: PRISMA 2020 manuscript checklist.

## References

[1] Masci C., Ciarrocchi I., Spadaro A., Necozione S., Marci M.C., Monaco A. (2013). Does orthodontic treatment provide a real functional improvement? a case control study. BMC Oral Health.

[2] Johal A., Alyaqoobi I., Patel R., Cox S. (2015). The Impact of Orthodontic Treatment on Quality of Life and Self-Esteem in Adult Patients. Eur. J. Orthod..

[3] Silvola A.S., Rusanen J., Tolvanen M., Pirttiniemi P., Lahti S. (2012). Occlusal Characteristics and Quality of Life Before and After Treatment of Severe Malocclusion. Eur. J. Orthod..

[4] Baldini A., Nota A., Cozza P. (2015). The association between Occlusion Time and Temporomandibular Disorders. J. Electromyogr. Kinesiol..

[5] Kecik D., Kocadereli I., Saatci I. (2007). Evaluation of the treatment changes of functional posterior crossbite in the mixed dentition. Am. J. Orthod. Dentofac. Orthop..

[6] Zhan Y., Yang M., Bai S., Zhang S., Huang Y., Gong F., Nong X. (2023). Effects of orthodontic treatment on masticatory muscles activity: A meta-analysis. Ann. Hum. Biol..

[7] Woźniak K., Piątkowska D., Lipski M., Mehr K. (2013). Surface electromyography in orthodontics—A literature review. Med. Sci. Monit..

[8] Ferrario V.F., Tartaglia G.M., Galletta A., Grassi G.P., Sforza C. (2006). The influence of occlusion on jaw and neck muscle activity: A surface EMG study in healthy young adults. J. Oral Rehabil..

[9] Castroflorio T., Bracco P., Farina D. (2008). Surface electromyography in the assessment of jaw elevator muscles. J. Oral Rehabil..

[10] Ngo C., Munoz C., Lueken M., Hülkenberg A., Bollheimer C., Briko A., Kobelev A., Shchukin S., Leonhardt S. (2022). A wearable, multi-frequency device to measure muscle activity combining simultaneous electromyography and electrical impedance myography. Sensors.

[11] Hugger S., Schindler H.J., Kordass B., Hugger A. (2012). Clinical relevance of surface EMG of the masticatory muscles. (Part 1): Resting activity, maximal and submaximal voluntary contraction, symmetry of EMG activity. Int. J. Comp. Dent..

[12] Szyszka-Sommerfeld L., Woźniak K., Matthews-Brzozowska T., Kawala B., Mikulewicz M., Machoy M. (2018). The electrical activity of the masticatory muscles in children with cleft lip and palate. Int. J. Paediatr. Dent..

[13] Klasser G.D., Okeson J.P. (2006). The clinical usefulness of surface electromyography in the diagnosis and treatment of temporomandibular disorders. J. Am. Dent. Assoc..

[14] Szyszka-Sommerfeld L., Machoy M., Lipski M., Woźniak K. (2019). The diagnostic value of electromyography in identifying patients with pain-related temporomandibular disorders. Front. Neurol..

[15] Szyszka-Sommerfeld L., Sycińska-Dziarnowska M., Spagnuolo G., Woźniak K. (2023). Surface electromyography in the assessment of masticatory muscle activity in patients with pain-related temporomandibular disorders: A systematic review. Front. Neurol..

[16] Woźniak K., Piątkowska D., Szyszka-Sommerfeld L., Buczkowska-Radlińska J. (2015). Impact of functional appliances on muscle activity: A surface electromyography study in children. Med. Sci. Monit..

[17] Saccucci M., Tecco S., Ierardoa G., Luzzi V., Festa F., Polimeni A. (2011). Effects of interceptive orthodontics on orbicular muscle activity: A surface electromyographic study in children. J. Electromyogr. Kinesiol..

[18] Lou T., Tran J., Castroflorio T., Tassi A., Cioffi I. (2021). Evaluation of masticatory muscle response to clear aligner therapy using ambulatory electromyographic recording. Am. J. Orthod. Dentofac. Orthop..

[19] Aggarwal P., Kharbanda O.P., Mathur R., Duggal R., Parkash H. (1999). Muscle response to the twin-block appliance: An electromyographic study of the masseter and anterior temporal muscles. Am. J. Orthod. Dentofac. Orthop..

[20] Mummolo S., Nota A., Tecco S., Caruso S., Marchetti E., Marzo G., Cutilli T. (2020). Ultra-low-frequency transcutaneous electric nerve stimulation (ULF-TENS) in subjects with craniofacial pain: A retrospective study. Cranio.

[21] Nishi S.E., Basri R., Alam M.K. (2016). Uses of electromyography in dentistry: An overview with meta-analysis. Eur. J. Dent..

[22] Spolaor F., Mason M., De Stefani A., Bruno G., Surace O., Guiotto A., Gracco A., Sawacha Z. (2020). Effects of Rapid Palatal Expansion on Chewing Biomechanics in Children with Malocclusion: A Surface Electromyography Study. Sensors.

[23] Dellavia C.P.B., Begnoni G., Zerosi C., Guenza G., Khomchyna N., Rosati R., Musto F., Pellegrini G. (2022). Neuromuscular Stability of Dental Occlusion in Patients Treated with Aligners and Fixed Orthodontic Appliance: A Preliminary Electromyographical Longitudinal Case-Control Study. Diagnostics.

[24] De Felício C.M., Sidequersky F.V., Tartaglia G.M., Sforza C. (2009). Electromyographic standardized indices in healthy Brazilian young adults and data reproducibility. J. Oral Rehabil..

[25] Svensson P., Wang K., Sessle B.J., Arendt-Nielsen L. (2004). Associations between pain and neuromuscular activity in the human jaw and neck muscles. Pain.

[26] Hugger A., Hugger S., Schindler H. (2008). Surface electromyography of the masticatory muscles for application in dental practice. Current evidence and future developments. Int. J. Comp. Dent..

[27] Nota A., Caruso S., Ehsani S., Ferrazzano G.F., Gatto R., Tecco S. (2021). Short-Term Effect of Orthodontic Treatment with Clear Aligners on Pain and sEMG Activity of Masticatory Muscles. Medicina.

[28] Paes-Souza S.A., Garcia M.A.C., Souza V.H., Morais L.S., Nojima L.I., Nojima M.D.C.G. (2023). Response of masticatory muscles to treatment with orthodontic aligners: A preliminary prospective longitudinal study. Dental Press J. Orthod..

[29] Nalamliang N., Thongudomporn U. (2023). Effects of class II intermaxillary elastics on masticatory muscle activity balance, occlusal contact area and masticatory performance: A multicenter randomised controlled trial. J. Oral Rehabil..

[30] Al-Dboush R., Al-Zawawi E., El-Bialy T. (2023). Does short-term treatment with clear aligner therapy induce changes in muscular activity?. Evid. Based Dent..

[31] Satygo E.A., Silin A.V., Ramirez-Yañez G.O. (2014). Electromyographic muscular activity improvement in Class II patients treated with the pre-orthodontic trainer. J. Clin. Pediatr. Dent..

[32] Michelotti A., Rongo R., Valentino R., D’Antò V., Bucci R., Danzi G., Cioffi I. (2019). Evaluation of masticatory muscle activity in patients with unilateral posterior crossbite before and after rapid maxillary expansion. Eur. J. Orthod..

[33] Page M.J., McKenzie J.E., Bossuyt P.M., Boutron I., Hoffmann T.C., Mulrow C.D., Shamseer L., Tetzlaff J.M., Akl E.A., Brennan S.E. (2021). The PRISMA 2020 statement: An updated guideline for reporting systematic reviews. BMJ.

[34] Sackett D.L., Strauss S.E., Richardson W.S., Rosenberg W., Haynes B.R. (2000). Evidence Based Medicine: How to Practice and Teach EBM.

[35] Stang A. (2010). Critical evaluation of the Newcastle-Ottawa scale for the assessment of the quality of nonrandomized studies in metaanalyses. Eur. J. Epidemiol..

[36] Sterne J.A.C., Savović J., Page M.J., Elbers R.G., Blencowe N.S., Boutron I., Cates C.J., Cheng H.Y., Corbett M.S., Eldridge S.M. (2019). RoB 2: A revised tool for assessing risk of bias in randomised trials. BMJ.

[37] Guyatt G., Oxman A.D., Akl E.A., Kunz R., Vist G., Brozek J., Norris S., Falck-Ytter Y., Glasziou P., DeBeer H. (2011). GRADE guidelines: 1. Introduction-GRADE evidence profiles and summary of findings tables. J. Clin. Epidemiol..

[38] Erdem A., Kilic N., Eröz B. (2009). Changes in soft tissue profile and electromyographic activity after activator treatment. Aust. Orthod. J..

[39] Martín C., Palma J.C., Alamán J.M., Lopez-Quiñones J.M., Alarcón J.A. (2012). Longitudinal evaluation of sEMG of masticatory muscles and kinematics of mandible changes in children treated for unilateral cross-bite. J. Electromyogr. Kinesiol..

[40] Ocak I., Soylu A.R., Aksu M. (2022). Changes in Orbicularis Oris Superior and Masseter Muscle Activities After Upper Incisor Protrusion in Class II Division 2 Malocclusion: An Electromyographic Study. Turk. J. Orthod..

[41] Petrović D., Vujkov S., Petronijević B., Šarčev I., Stojanac I. (2014). Examination of the bioelectrical activity of the masticatory muscles during Angle’s Class II division 2 therapy with an activator. Vojnosanit. Pregl..

[42] Piancino M.G., Falla D., Merlo A., Vallelonga T., de Biase C., Dalessandri D., Debernardi C. (2016). Effects of therapy on masseter activity and chewing kinematics in patients with unilateral posterior crossbite. Arch. Oral Biol..

[43] Uysal T., Yagci A., Kara S., Okkesim S. (2012). Influence of pre-orthodontic trainer treatment on the perioral and masticatory muscles in patients with Class II division 1 malocclusion. Eur. J. Orthod..

[44] Wasinwasukul P., Nalamliang N., Pairatchawan N., Thongudomporn U. (2022). Effects of anterior bite planes fabricated from acrylic resin and thermoplastic material on masticatory muscle responses and maximum bite force in children with a deep bite: A 6-month randomised controlled trial. J. Oral Rehabil..

[45] Yuen S.W., Hwang J.C., Poon P.W. (1990). Changes in power spectrum of electromyograms of masseter and anterior temporal muscles during functional appliance therapy in children. Am. J. Orthod. Dentofac. Orthop..

[46] Kiliaridis S., Mills C., Antonarakis G. (2010). Masseter muscle thickness as a predictive variable in treatment outcome of the twin- block appliance and masseteric thickness changes during treatment. Orthod. Craniofac. Res..

[47] Antonarakis G., Kiliaridis S. (2015). Predictive value of masseter muscle thickness and bite force on class II functional appliance treatment: A prospective controlled study. Eur. J. Orthod..

[48] Tomonari H., Kubota T., Yagi T., Kuninori T., Kitashima F., Uehara S., Miyawaki S. (2014). Posterior scissors-bite: Masticatory jaw movement and muscle activity. J. Oral Rehabil..

[49] English J.D., Buschang P.H., Throckmorton G.S. (2002). Does malocclusion affect masticatory performance?. Angle Orthod..

[50] Alarcón J.A., Martín C., Palma J.C. (2000). Effect of unilateral posterior crossbite on the electromyographic activity of human masticatory muscles. Am. J. Orthod. Dentofac. Orthop..

[51] Alarcón J.A., Martín C., Palma J.C., Menendez-Nunez M. (2009). Activity of jaw muscles in unilateral cross-bite without mandibular shift. Arch. Oral Biol..

[52] Bakke M., Michler L., Moller E. (1992). Occlusal control of mandibular elevator muscles. Scand. J. Dent. Res..

[53] Bakke M., Moller E. (1992). Craniomandibular disorders and masticatory muscle function. Scand. J. Dent. Res..

[54] Dimberg L., Arnrup K., Bondemark L. (2015). The impact of malocclusion on the quality of life among children and adolescents: A systematic review of quantitative studies. Eur. J. Orthod..

[55] Alshammari A., Almotairy N., Kumar A., Grigoriadis A. (2022). Effect of malocclusion on jaw motor function and chewing in children: A systematic review. Clin. Oral Investig..

[56] Janson G., Branco N.C., Fernandes T.M.F., Sathler R., Garib D., Lauris J.R.P. (2011). Influence of orthodontic treatment, midline position, buccal corridor and smile arc on smile attractiveness. Angle Orthod..

[57] de Paula J.D.F., Santos N.C.M., da Silva É.T., Nunes M.F., Leles C.R. (2009). Psychosocial impact of dental esthetics on quality of life in adolescents. Angle Orthod..

[58] Stocka A., Kuc J., Sierpinska T., Golebiewska M., Wieczorek A. (2015). The Influence of Emotional State on the Masticatory Muscles Function in the Group of Young Healthy Adults. BioMed Res. Int..

[59] Zieliński G., Ginszt M., Zawadka M., Rutkowska K., Podstawka Z., Szkutnik J., Majcher P., Gawda P. (2021). The Relationship between Stress and Masticatory Muscle Activity in Female Students. J. Clin. Med..

[60] Zieliński G., Suwała M., Ginszt M., Szkutnik J., Majcher P. (2018). Bioelectric activity of mastication muscles and the functional impairment risk groups concerning the masticatory muscles. Acta Bioeng. Biomech..

[61] Augusto V.G., Perina K.C.B., Penha D.S.G., Dos Santos D.C.A., Oliveira V.A.S. (2016). Temporomandibular Dysfunction, Stress and Common Mental Disorder in University Students. Acta Ortop. Bras..

[62] Castroflorio T., Talpone F., Deregibus A., Piancino M.G., Bracco P. (2004). Effects of a functional appliance on masticatory muscles of young adults suffering from muscle-related temporomandibular disorders. J. Oral Rehabil..

[63] Patil S.R., Doni B.R., Patil C., Nawab S., Khursheed Alam M. (2023). Role of Electromyography in Dental Research: A Review. J. Res. Dent. Maxillofac. Sci..

[64] Ferrario V.F., Sforza C., Colombo A., Ciusa V. (2000). An electromyographic investigation of masticatory muscles symmetry in normo-occlusion subjects. J. Oral Rehabil..

